# Predicting Retinopathy of Prematurity in Thailand: A Practical Model for Risk-Based Screening

**DOI:** 10.3390/diagnostics16193207

**Published:** 2026-10-02

**Authors:** Suppachai Lawanaskol, Jayanton Patumanond, Phichayut Phinyo, Songklot Noppakaonamchokchai, Chompoonoot Kunaviktikul, Anavat Bupphachareonsuk, Pakawadi Vuttipittayamongkol, Apichat Tantraworasin, Janejit Choovuthayakorn

**Affiliations:** 1 Center for Clinical Epidemiology and Clinical Statistics, Department of Biomedical Informatics and Clinical Epidemiology (BioCE), Faculty of Medicine, Chiang Mai University, Chiang Mai 50200, Thailand; 2 Chaiprakarn Hospital, Chiang Mai 50320, Thailand; 3 Clinical Epidemiology Unit, Faculty of Medicine, Naresuan University, Phitsanulok 65000, Thailand; 4 Department of Ophthalmology, Chiang Rai Prachanukroh Hospital, Chiang Rai 57000, Thailand; 5 Bangkok Hospital Chiang Mai, Chiang Mai 50000, Thailand; 6 Department of Pediatrics, Lampang Hospital, Lampang 52000, Thailand; 7 Clinical Surgical Research Center, Faculty of Medicine, Chiang Mai University, Chiang Mai 50200, Thailand; 8 Department of Surgery, Faculty of Medicine, Chiang Mai University, Chiang Mai 50200, Thailand; 9 Department of Ophthalmology, Faculty of Medicine, Chiang Mai University, Chiang Mai 50200, Thailand

**Keywords:** retinopathy of prematurity, infant, premature, clinical decision rules, risk assessment

## Abstract

**Background/Objectives**: Retinopathy of prematurity (ROP) screening must balance timely detection with feasibility, particularly in resource-limited settings. We aimed to develop a pragmatic, locally derived prediction model to support prioritization of ophthalmologic assessment among Thai neonates already eligible for ROP screening. **Methods**: We conducted a retrospective cohort study of neonates undergoing their first planned ROP screening examination at Chiang Rai Prachanukroh Hospital (2011–2015) and Lampang Hospital (2021–2022). Prespecified predictors included gestational age, birth weight, multiple pregnancy, respiratory distress syndrome, neonatal anemia, neonatal sepsis, necrotizing enterocolitis, bronchopulmonary dysplasia, duration of oxygen exposure, and duration of mechanical ventilation. A multivariable logistic regression model was developed. Performance was evaluated using discrimination, calibration, decision curve analysis, and bootstrap internal validation with 1000 resamples. **Results**: Among 681 infants, 50 (7.3%) had any ROP detected at the index examination. The apparent area under the receiver operating characteristic curve was 0.901 (95% CI, 0.856–0.946), and the optimism-corrected estimate was 0.883 (bootstrap 95% CI, 0.841–0.928). Optimism-corrected calibration-in-the-large was 0.008, the expected-to-observed ratio was 1.005, and the calibration slope was 0.866. At the sensitivity-constrained exploratory cut-off of 1.5%, sensitivity was 96.0% and specificity was 53.4%. The observed ROP risk was 14.0% in the higher-risk group and 0.6% in the lower-risk group. Decision curve analysis suggested net benefit across threshold probabilities up to 0.20. **Conclusions**: The model showed good discrimination and may support risk stratification and prioritization among infants already eligible for ROP screening. The 1.5% cut-off remains exploratory, and independent external validation, preferably in prospective contemporary cohorts, is required before clinical implementation.

## 1. Introduction

Retinopathy of prematurity (ROP) is a leading cause of preventable childhood blindness worldwide, resulting from abnormal retinal vascular development in preterm infants. More broadly, preterm birth is associated with neurological, cognitive, and sensory morbidity, highlighting the wider developmental vulnerability of this population [1]. Early detection and timely intervention are essential to preserve vision; however, effective screening requires balancing sensitivity with workload feasibility [2,3,4]. Standard international screening guidelines—typically based on birth weight below 1500 g or gestational age under 30 weeks—identify most high-risk infants but may overlook ROP cases among heavier or more mature neonates [5,6,7,8]. Conversely, universal screening irrespective of risk factors can overburden ophthalmology services, particularly in resource-limited settings such as provincial hospitals in Thailand.

Numerous prediction and screening models, including ROPScore, CHOP-ROP, WINROP, G-ROP, DIGIROP, and STEP-ROP, have been developed to improve the efficiency of ROP screening by incorporating perinatal and postnatal risk information [9,10,11,12]. Several of these approaches have subsequently been evaluated in Asian populations, including Thailand. In Thailand, the G-ROP screening criteria have been externally evaluated, and risk-factor-based models for severe ROP have also been developed in preterm infants [13,14]. Subsequent commentary has highlighted the need to consider temporal changes in clinical practice and further validation when interpreting such models [15]. These studies support the feasibility of risk-based approaches but also highlight differences across models in target populations, endpoint definitions, prediction timing, predictor availability, and intended clinical use. Accordingly, the present study sought to develop a pragmatic model based on routinely available clinical variables in the participating hospitals, rather than to imply that a locally developed model is inherently superior to existing approaches [11].

The present study focused on any ROP detected at the first planned ophthalmologic screening examination because the intended use was risk stratification at the initial screening encounter rather than prediction of subsequent progression to severe or treatment-requiring ROP.

This study aimed to develop a pragmatic prediction model using routinely available clinical variables to estimate the probability of any ROP being detected at the first planned ophthalmologic screening examination among infants already eligible for ROP screening. The model was intended for exploratory risk stratification rather than to replace established screening eligibility criteria or exclude infants from ophthalmologic examination.

## 2. Materials and Methods

### 2.1. Study Design and Data Source

We conducted a retrospective cohort study to develop a multivariable prediction model for any ROP detected at the first planned ophthalmologic screening examination among neonates undergoing routine ROP screening. The study was conducted at Chiang Rai Prachanukroh Hospital during 2011–2015 and at Lampang Hospital during 2021–2022.

### 2.2. Inclusion Criteria/Exclusion Criteria

Eligible participants were neonates who met institutional or national indications for ROP screening within the first 28 postnatal days. The first ophthalmologic examination was performed when the infant was considered clinically stable; therefore, the examination could occur after 28 days of postnatal age. Infants were included if they had a documented result from their first planned ROP screening examination. Infants were excluded if (i) the eye examination was performed for reasons other than any ROP screening, or (ii) any ROP had been previously diagnosed before the index screening episode (i.e., not a first-time screening for any ROP).

### 2.3. Data Collection

Newborns with any documented ROP screening results, either positive or negative for any ROP, were retrospectively reviewed. Demographic, perinatal, and clinical data prior to any ROP screening were retrieved from medical records and recorded using a standardized pre-screening assessment form. The collected data were systematically entered and analyzed to develop a prediction model for estimating the probability of any ROP being detected at the first planned screening examination. Clinical and perinatal data were retrospectively retrieved from the medical records. Although screening eligibility was established within the first 28 postnatal days, the timing of the first ophthalmologic examination varied according to the infant’s clinical stability. Consequently, cumulative predictors such as oxygen exposure and mechanical ventilation could extend beyond 28 days and varied according to the timing of the first planned ophthalmologic examination.

### 2.4. Candidate Predictors

Candidate predictors were selected a priori based on clinical relevance and availability up to the defined prediction time, and were recorded using a standardized pre-screening assessment form. Predictors included:

Sex (male/female)

Gestational age (days)

Birth weight (grams)

Multiple pregnancy (yes/no)

Respiratory distress syndrome (RDS) (yes/no)

Neonatal anemia (yes/no)

Neonatal sepsis (yes/no)

Necrotizing enterocolitis (NEC) (yes/no)

Bronchopulmonary dysplasia (BPD) (yes/no)

Oxygen exposure (days)

Mechanical ventilation (days)

Early-life predictors—including sex, gestational age, multiple pregnancy, and respiratory distress syndrome—were assessed based on clinical diagnoses made within the first few days after delivery, upon admission to the neonatal intensive care unit (NICU) [9]. Time-varying (follow-up) predictors—including neonatal anemia, neonatal sepsis, necrotizing enterocolitis, bronchopulmonary dysplasia, duration of oxygen exposure (days), and duration of mechanical ventilation (days)—were ascertained using information available up to the defined prediction time, when the infant was considered sufficiently stable and the first ophthalmologic examination was requested or scheduled [11]. Sex was initially evaluated but was not retained because it provided negligible incremental predictive information.

Bronchopulmonary dysplasia was treated as a time-varying clinical predictor and was coded according to the diagnosis documented in the neonatal medical record by the defined prediction time. Infants without a documented BPD diagnosis at that time were classified as not having BPD, regardless of whether BPD was subsequently diagnosed later during hospitalization. BPD was defined based on the clinical diagnosis documented by the treating neonatologist in the medical record, using routine clinical practice at each participating hospital.

Continuous predictors were retained on their continuous scales without arbitrary categorization. Potential non-linearity was assessed by adding quadratic terms for birth weight, gestational age, duration of oxygen exposure, and duration of mechanical ventilation. The model containing linear terms only was compared with the model containing both linear and quadratic terms. Because the quadratic specification did not materially improve model discrimination, the simpler linear specification was retained in the final model [16].

### 2.5. Prediction Horizon and Moment of Prediction

The prediction time was defined as the clinical time point at which the infant was considered sufficiently stable to undergo the first planned ROP examination. In routine practice at the participating hospitals, ophthalmologic examination was generally performed on the same day or within the next 1–2 days after the infant was considered clinically stable. Therefore, for model development, the prediction time and the first ophthalmologic examination were considered essentially contemporaneous clinical time points. Predictor information, including cumulative oxygen exposure and mechanical ventilation duration, was based on information available up to this clinical time point. The exact interval between the clinical decision that the infant was stable and the actual ophthalmologic examination was not systematically recorded.

### 2.6. Endpoint Measurement

Retinopathy of prematurity status was determined by an ophthalmologist at the first planned screening examination, which was performed after the infant was considered clinically stable, and was documented in the medical record. The primary endpoint was any ROP detected at the first planned ophthalmologic screening examination (index examination), defined as ROP of any stage detected in either eye. Subsequent disease progression, spontaneous regression, treatment, or ROP diagnosed after the index examination did not alter the endpoint classification. Infants were referred for screening by neonatologists or pediatricians according to predefined institutional or national eligibility criteria, and ophthalmology consultation was requested once the infant was considered clinically stable enough to undergo examination. Infants whose eye examination was performed for an indication other than planned ROP screening, including incidental or non-screening examinations, were excluded. Infants with any ROP diagnosed before the index screening examination were also excluded.

ROP status was subsequently determined by the ophthalmologist during the first planned examination at this clinical time point.

### 2.7. Study Size Estimation

Based on the C-statistic of 0.93 reported by Pivodic et al. [10], the sample-size calculation assumed 10 predictor parameters, an anticipated ROP prevalence of 5%, and a target shrinkage factor of 0.90. Under these assumptions, a minimum sample size of 556 neonates, including approximately 28 ROP events, was required [11].

### 2.8. Missing Data Handling

According to intensive care unit routine data, there are no missing data. Data completeness was assessed during data abstraction by reviewing the required study variables against the original neonatal and ophthalmologic records. Records with initially incomplete entries were rechecked against the source medical records. No missing values remained for the variables included in the analytic dataset.

### 2.9. Model Development

We developed a multivariable prediction model for the binary outcome (any ROP yes/no) using logistic regression. All prespecified candidate predictors were entered simultaneously. Face validity of predictor effects was assessed using clinical plausibility and statistical evidence—specifically the direction and magnitude of effect estimates with corresponding confidence intervals—rather than statistical significance alone.

For continuous predictors, alternative functional forms (e.g., linear vs. non-linear specifications) were evaluated by comparing model performance. When performance was comparable, we conservatively selected the simplest specification to minimize overfitting and enhance clinical interpretability [16]. Potential non-linearity was assessed by adding quadratic terms for birth weight, gestational age, oxygen exposure duration, and mechanical ventilation duration. Continuous predictors were expressed in the same units in both the linear and quadratic models. The AuROCs of the two models were compared using DeLong’s test for correlated ROC curves.

We deployed the prediction model as an online prognostic index application.

### 2.10. Model Performance

We assessed:

- Discrimination: C-statistic (AuROC) with 95% CI.
- Calibration: calibration-in-the-large, calibration slope, and calibration plot [17].
- Clinical decision net benefit analysis: using clinical decision curve [18,19].

### 2.11. Model Presentation, Web-Based Implementation and Risk Classification

To facilitate clinical use, the final logistic regression model was implemented as a web-based calculator that estimates the individual predicted probability of any ROP. The calculator is publicly available at https://suppachai-lawanaskol.github.io/rop/ (accessed on 29 September 2026). For exploratory risk stratification, we selected the highest predicted-risk cut-off that maintained a sensitivity of at least 95% for any ROP detected at the index examination. Because the development cohort contained 50 ROP events, attainable sensitivity values changed in increments of 2%; this criterion corresponded to a sensitivity of 96% and a predicted-risk cut-off of 1.5%. Infants with predicted risk below 1.5% were classified as lower risk, whereas those with predicted risk of 1.5% or greater were classified as higher risk. This sensitivity-constrained cut-off was intended to support exploratory risk stratification and prioritization among infants already eligible for ROP screening and was not intended to determine screening eligibility or to exclude lower-risk infants from established screening.

### 2.12. Internal Validation

Internal validation was performed using 1000 bootstrap resamples to quantify optimism and obtain optimism-corrected estimates of overall performance, discrimination, and calibration [20].

### 2.13. Sensitivity Analysis Using Penalized Regression

As a sensitivity analysis, ridge-penalized logistic regression was fitted using the same prespecified predictor set as the primary logistic regression model. Ridge penalization was selected because the principal concern was coefficient instability and potential overfitting rather than variable selection. The penalty parameter was selected using 10-fold cross-validation. Predictive performance of the ridge model was compared with that of the standard logistic regression model in terms of discrimination, calibration, and overall predictive accuracy. Differences in AuROC were assessed using DeLong’s test for correlated ROC curves. Calibration-in-the-large and calibration slope were estimated from the predicted probabilities, and overall predictive accuracy was assessed using the Brier score. Ridge-penalized logistic regression was implemented using the elastic-net framework with the mixing parameter fixed at zero (alpha = 0).

### 2.14. Statistical Analysis

Continuous variables were compared between infants with and without ROP using the independent-samples *t*-test for approximately normally distributed variables and the Wilcoxon rank-sum test for non-normally distributed variables. Categorical variables were compared using the exact probability test. All statistical tests were two-sided, and a *p* value < 0.05 was considered statistically significant. Statistical analyses were performed using Stata/SE 19.5 (StataCorp LLC, College Station, TX, USA).

## 3. Results

**Figure 1:**
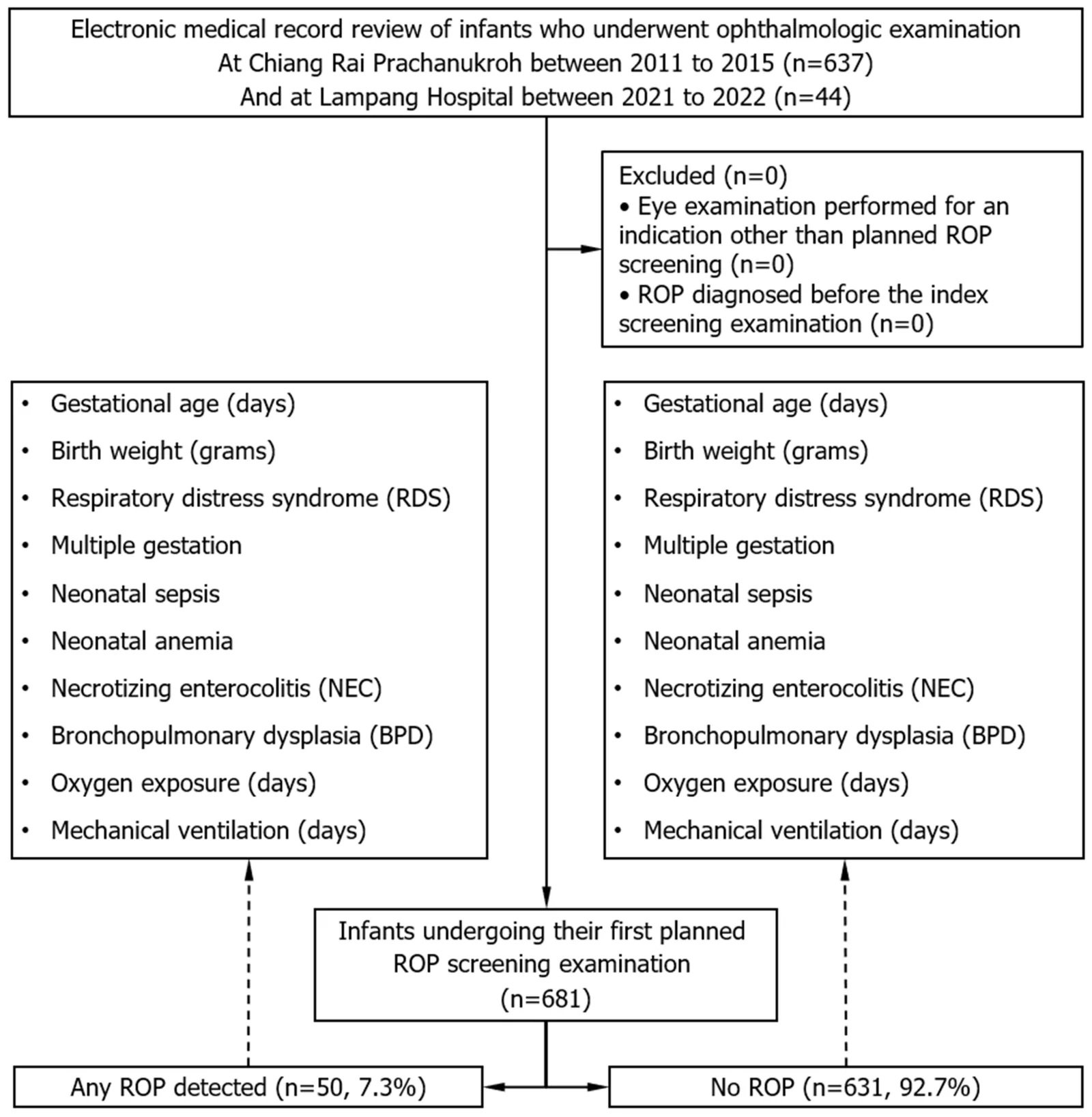
Study flow diagram.

A total of 681 neonates were included, comprising 44 (6.5%) from Lampang Hospital and 637 (93.5%) from Chiang Rai Prachanukroh Hospital. Overall, any ROP was detected at the index screening examination in 50 infants (7.3%), whereas 631 (92.7%) were not. All predictors were obtained from routinely collected clinical data, and no missing values were observed (Figure 1).

Infants with any ROP had lower birth weight and gestational age, longer durations of oxygen exposure and mechanical ventilation, and higher frequencies of RDS, BPD, neonatal sepsis, and neonatal anemia. Sex, multiple pregnancy, and NEC did not differ significantly between groups (Table 1).

For model development, multivariable logistic regression under a linearity assumption identified birth weight and gestational age as significant predictors of ROP, whereas other covariates were not statistically significant in the fitted model. Testing non-linearity using a quadratic specification for continuous predictors did not yield a statistically meaningful improvement in discrimination (*p* = 0.200 by DeLong’s test). Therefore, the simpler linear specification was retained (Table 2; Supplementary Figure S1).

**Figure 2:**
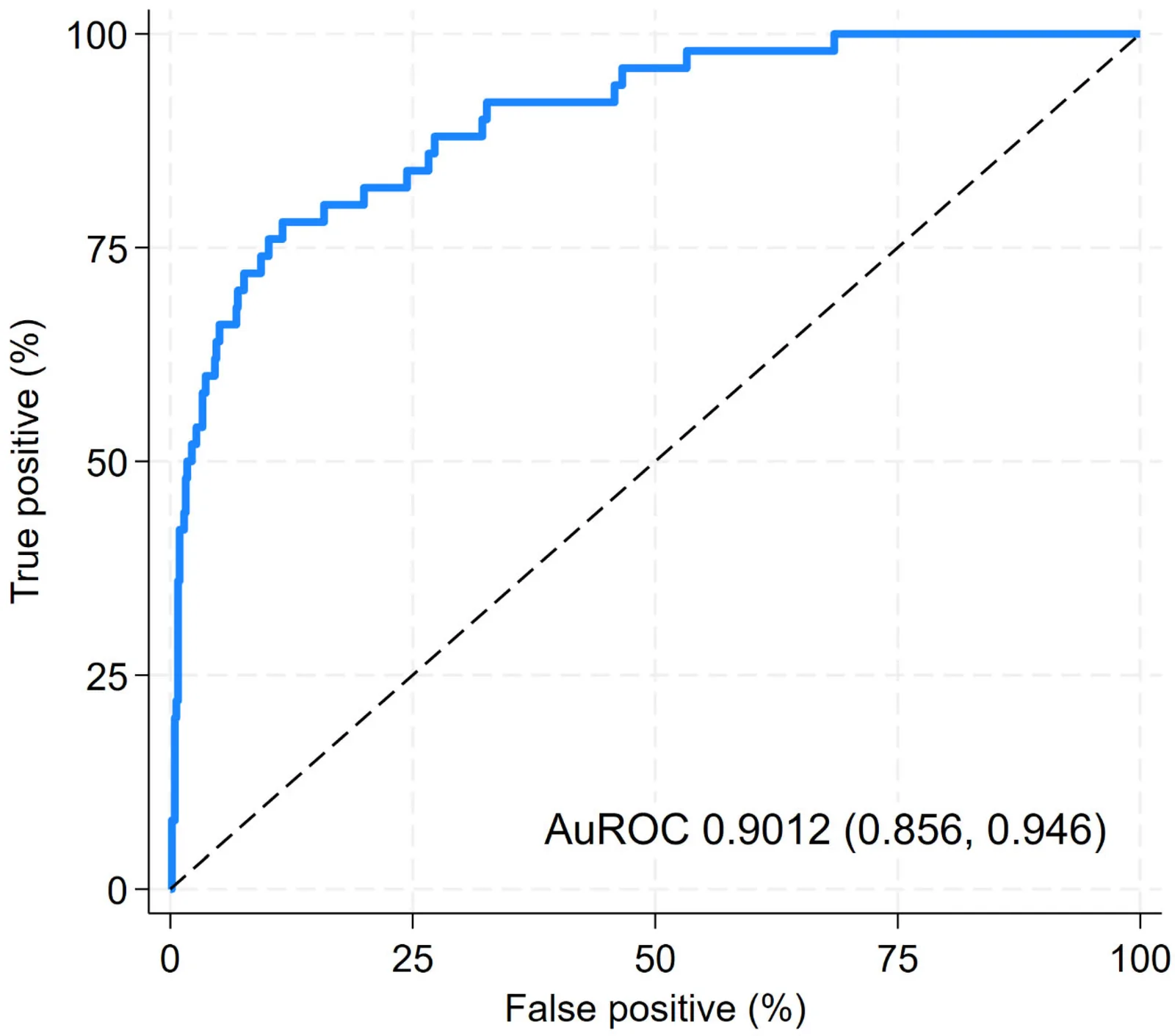
Receiver operating characteristic (ROC) curve.

**Figure 3:**
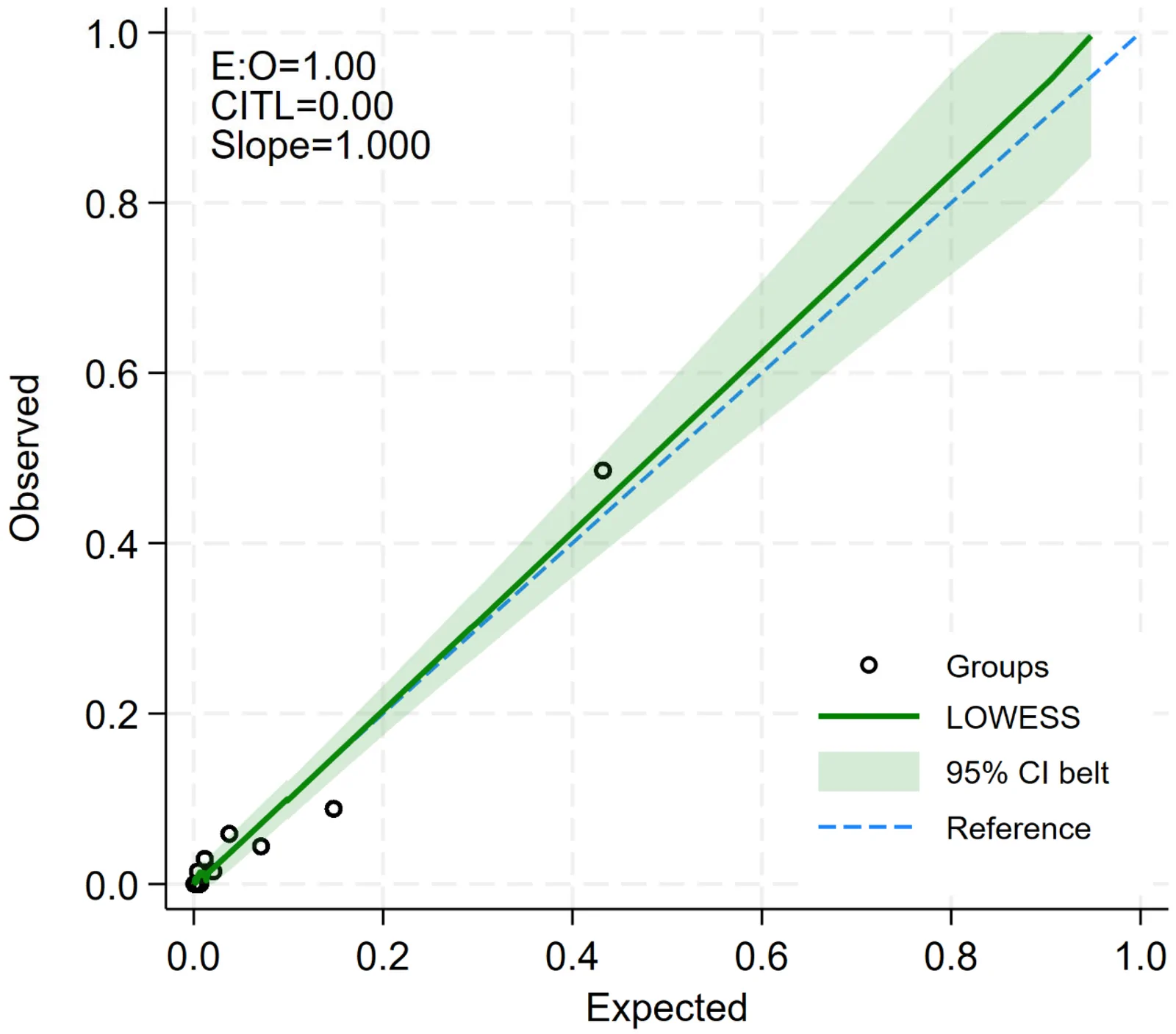
Calibration plot.

The model demonstrated good discrimination, with an area under the receiver operating characteristic curve (AuROC) of 0.901 (95% CI, 0.856–0.946) (Figure 2). In the development dataset, apparent calibration estimates were a CITL of 0.00, an E:O ratio of 1.00, and a calibration slope of 1.00 (Figure 3).

**Figure 4:**
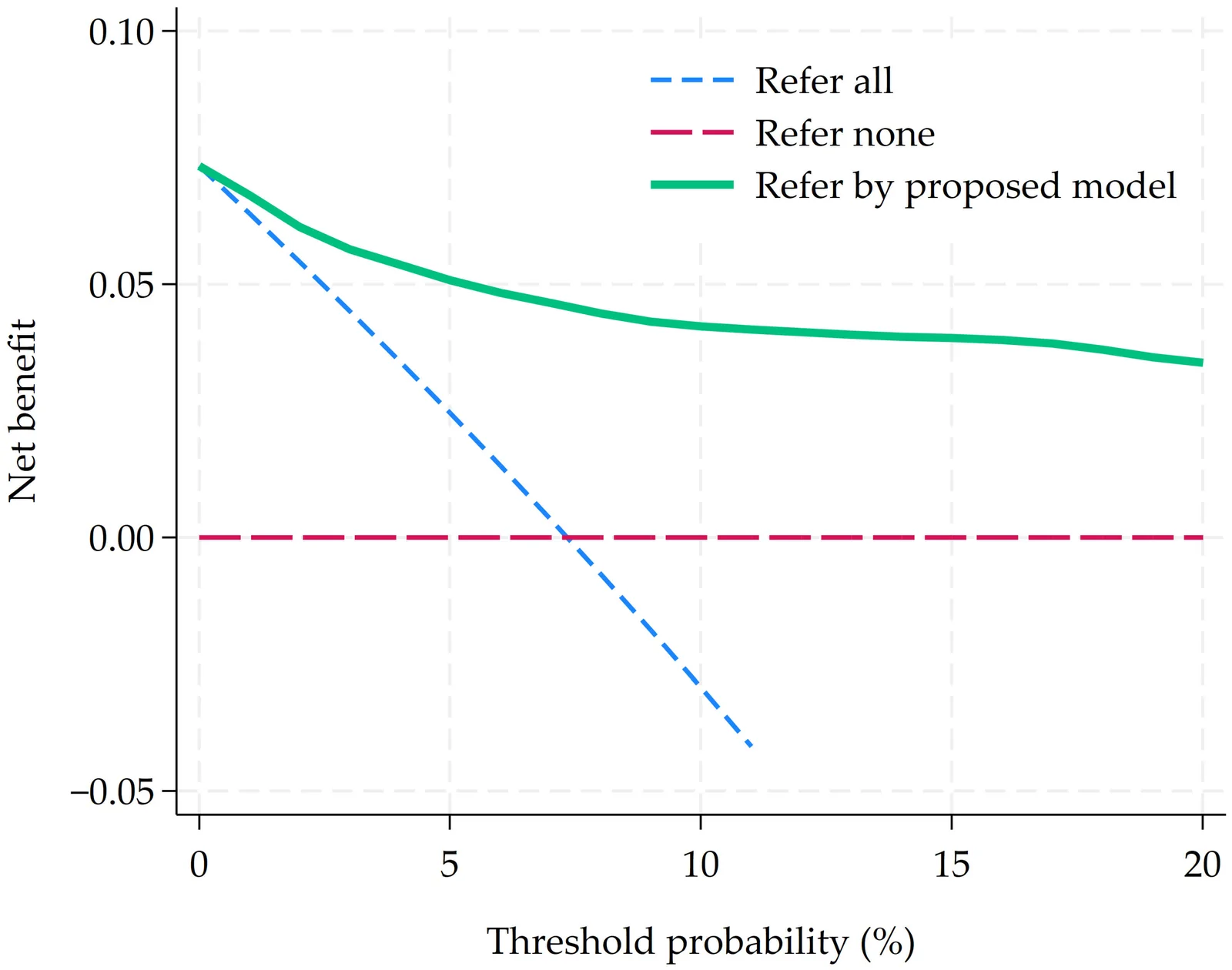
Decision curve analysis.

Clinical utility was assessed using decision curve analysis. The model provided greater net benefit up to a threshold probability of 0.20; beyond this threshold, the incremental benefit diminished (Figure 4).

The positive predictive value was 14.0% (95% CI, 10.5–18.2), the negative predictive value was 99.4% (95% CI, 97.9–99.9), the positive likelihood ratio was 2.06 (95% CI, 1.86–2.28), and the negative likelihood ratio was 0.07 (95% CI, 0.02–0.29).

In an exploratory center-stratified analysis of apparent discrimination, the AuROC was 0.882 (95% CI, 0.827–0.937) at Chiang Rai Prachanukroh Hospital and 0.898 (95% CI, 0.801–0.996) at Lampang Hospital, with no statistical evidence of a difference between centers (*p* = 0.878) (Supplementary Figure S2).

Bootstrap internal validation using 1000 resamples yielded an optimism-corrected C-statistic of 0.883 (95% CI, 0.841–0.928), an optimism-corrected scaled Brier score of 26.1%, an expected-to-observed ratio of 1.005 (95% CI, 0.795–1.247), calibration-in-the-large of 0.008 (95% CI, −0.374 to 0.385), and a calibration slope of 0.866 (95% CI, 0.606–1.129). The bootstrap shrinkage factor was 0.866 (Table 3).

At the sensitivity-constrained cut-off of 1.5%, 342 of 681 infants (50.2%) were classified as higher risk, including 48 of the 50 infants (96.0%) with any ROP detected at the index examination. Sensitivity was 96.0% (95% CI, 86.3–99.5) and specificity was 53.4% (95% CI, 49.4–57.4). The observed ROP risk was 14.0% in the higher-risk group and 0.6% in the lower-risk group (Table 4).

The primary model was retained as prespecified, while ridge-penalized logistic regression was additionally performed as a sensitivity analysis. The ridge model showed nearly identical discrimination to the standard logistic model (AuROC 0.900 vs. 0.901; DeLong *p* = 0.819), with similar overall predictive accuracy. We therefore did not replace the prespecified primary model post hoc with the penalized model (Supplementary Table S1).

## 4. Discussion

### 4.1. Principal Findings

In this two-center cohort of 681 neonates, we developed a pragmatic prediction model for any ROP detected at the first planned ophthalmologic screening examination using routinely collected clinical data.

After internal validation using 1000 bootstrap resamples, the model retained good discrimination, with an optimism-corrected AUROC of 0.883. Optimism-corrected calibration-in-the-large and the expected-to-observed ratio were close to their ideal values (CITL, 0.008; E:O ratio, 1.005). However, the optimism-corrected calibration slope was 0.866, suggesting some overfitting, although its bootstrap confidence interval was wide (0.606–1.129). Decision curve analysis suggested net clinical benefit up to a threshold probability of 0.20, supporting potential usefulness for screening decision-making [6,18].

### 4.2. Interpretation and Comparison with Existing Evidence

The model’s performance is broadly comparable to other clinically oriented any ROP prediction tools that rely on readily available predictors. Birth weight and gestational age were statistically significant predictors in the multivariable model, consistent with established ROP epidemiology. We also incorporated duration of oxygen supplementation as a practical measure of cumulative exposure; unlike predictors that fluctuate or “improve” over time, cumulative oxygen exposure is monotonic and clinically interpretable, which may facilitate practical application in routine clinical settings [12,21,22].

We intentionally avoided relying on modifiable predictors based solely on their current status at a given time point, while retaining the history of clinically relevant events such as neonatal sepsis and anemia, because (i) current clinical conditions can resolve with treatment while prior biological insult may already have occurred, and (ii) the timing of ascertainment varies across settings and may lead to misclassification. Although prolonged oxygen exposure remains biologically relevant to retinal vascular injury, its coefficient in a multivariable prediction model should not be interpreted as estimating an independent causal effect [22,23].

### 4.3. Clinical Usefulness and Intended Application

At the sensitivity-constrained exploratory cut-off of 1.5%, approximately half of the cohort was classified as higher risk, including 96% of infants with any ROP detected at the index examination. The observed ROP risk was 14.0% in the higher-risk group compared with 0.6% in the lower-risk group. This threshold was selected to prioritize sensitivity while retaining as much specificity as possible; however, two ROP cases remained below the cut-off. Accordingly, the model should not be used to exclude otherwise eligible infants from screening or to replace established screening criteria. Infants classified as lower risk should continue the standard ROP screening schedule, and lower predicted risk should not be used as a reason to delay screening. Its potential clinical role is more appropriately considered as risk stratification and prioritization among infants who already meet screening eligibility. Decision curve analysis suggested greater net benefit for the proposed model than the alternative strategies across threshold probabilities up to 0.20. The 1.5% cut-off remains exploratory and requires independent external validation before being considered for clinical implementation. Importantly, threshold selection should be context-specific, reflecting local prevalence, available screening capacity, and tolerance for missed cases [18]. The model identified a subgroup with a low observed probability of any ROP but should not be used to exclude otherwise eligible infants from screening.

The distinction between any ROP and clinically severe ROP is important when considering the intended use of the model. Type 1 ROP and treatment-requiring ROP represent more clinically consequential endpoints and are directly relevant to treatment decisions, whereas the present model was developed to estimate the probability of any ROP being detected at the first planned screening examination. Accordingly, the model should not be interpreted as a predictor of progression to Type 1 ROP or the subsequent need for treatment, and its performance cannot be extrapolated to these outcomes. Models specifically targeting severe or treatment-requiring ROP may have greater relevance for treatment-oriented decision-making. Future studies with prospectively and consistently documented ROP severity, plus disease, longitudinal progression, and treatment outcomes should evaluate prediction of these clinically important endpoints.

### 4.4. Model Performance Across Centers

Because both centers contributed to model development, these estimates do not represent external validation. The center-stratified analysis was exploratory. Although apparent discrimination was similar across the two participating centers, the Lampang estimate was based on only 44 infants and was therefore imprecise. These findings should not be interpreted as evidence of equivalent performance or transportability across centers. Differences in referral policies, screening practices, case mix, and clinical management may contribute to variation in model performance across settings. Future studies may evaluate explicit center effects or recalibration strategies when implementing the model in new settings [24].

Notably, the consultation policy with ophthalmologists changed after completion of the Chiang Rai Prachanukroh cohort (*n* = 637). This local cohort likely influenced neonatologists’ consultation behavior (i.e., the decision threshold for referral), leading to a shift in the screening/consultation pathway over time. Although apparent discrimination was similar across the participating centers, the substantial differences in calendar period and clinical practice preclude conclusions regarding temporal stability. Calibration may drift over time, and contemporary external validation will therefore be required before implementation [25].

### 4.5. Strengths

A strength of this study is the use of routinely available and clinically interpretable predictors, which may facilitate pragmatic implementation after appropriate external validation. Bootstrap internal validation provided optimism-corrected performance estimates and demonstrated some overfitting [26].

### 4.6. Limitations

Several limitations should be acknowledged.

First, the number of any ROP events was limited (*n* = 50), which restricted the effective information available for estimating multiple predictor coefficients and increased the risk of coefficient instability and overfitting. Although the prespecified sample-size criterion was met and bootstrap internal validation was performed, these procedures do not eliminate uncertainty arising from the limited number of events [26,27].

Second, although quadratic terms did not improve discrimination, other non-linear functional forms could influence calibration; nonetheless, simplicity and feasibility were prioritized [16].

Third, although the dataset included more recent observations from the past 5 years, a substantial proportion of the events required to provide adequate statistical power came from earlier calendar periods [15,27]. This raises the possibility of temporal changes in clinical practice, oxygen management, and screening behavior. However, we did not observe sufficient heterogeneity to justify excluding older data entirely, and retaining these data was considered necessary to preserve the effective number of outcome events.

Fourth, the outcome was defined as any ROP rather than type 1 ROP. In earlier records, type 1 ROP, plus disease, treatment-requiring ROP, and other detailed ophthalmologic features were not consistently documented; therefore, reliable modeling of these more clinically consequential endpoints was not feasible. This limits the direct applicability of the model to treatment-oriented decision-making.

Fifth, postnatal weight gain was unavailable in the retrospective dataset. Although early postnatal weight gain has been incorporated into some ROP prediction approaches, it was not routinely recorded in a structured format and was not consistently used by neonatologists as a clinical risk factor in this setting. Its absence may therefore have limited predictive performance, particularly for models intended to capture postnatal disease evolution [14,21].

Sixth, although the sensitivity-constrained cut-off of 1.5% identified 96% of infants with any ROP at the index examination, two ROP cases remained below the cut-off. This threshold was derived from the development cohort and has not been externally validated; therefore, it should not be interpreted as a definitive clinical decision threshold or used to exclude infants from screening. Any future implementation threshold should be evaluated in independent populations and should reflect screening capacity, the consequences of missed cases, and the intended clinical action.

Seventh, several predictors showed coefficient directions that differed from their expected marginal associations. These estimates should not be interpreted as indicating protective effects because birth weight, gestational age, respiratory support, and neonatal morbidities are interrelated markers of prematurity and illness severity. Simultaneous multivariable analysis may therefore have produced multicollinearity, suppression, and effect direction reversal [28]. The limited number of ROP events, sparse data for some binary predictors, restriction of the cohort to infants who had already undergone screening, and overlapping information between oxygen exposure and mechanical ventilation may have further contributed to coefficient instability. Together with an optimism-corrected calibration slope of 0.866, these findings suggest some overfitting [20,26,27].

Eighth, an additional limitation concerns the timing of prediction. The prediction moment was not anchored to a fixed postnatal age or postmenstrual age but instead occurred when the treating neonatologist considered the infant sufficiently stable for the first ophthalmologic examination. Consequently, cumulative predictors such as duration of oxygen exposure and mechanical ventilation may reflect both underlying illness severity and the elapsed time until screening. This variable prediction time limits interpretation of the model as a prospective tool for determining screening timing or prioritization. Future models intended for this purpose should use a prespecified clinical landmark with a clearly defined prediction horizon.

Ninth, because the first ophthalmologic examination could occur before a formal diagnosis of BPD was established, BPD may have been underascertained at the prediction time in some infants. However, later diagnoses were not retrospectively incorporated into the predictor status, thereby preserving temporal validity.

Tenth, exact postnatal age and postmenstrual age at the first examination, as well as the individual interval between determination of clinical stability and ophthalmologic examination, were not systematically recorded. However, in routine practice, the examination was generally performed on the same day or within 1–2 days once the infant was considered clinically stable.

Finally, the cohort was derived from two centers with imbalanced contributions across hospitals, which may limit generalizability. Model performance, particularly calibration, may vary across settings because of differences in case mix, neonatal care practices, oxygen management protocols, screening criteria, and calendar periods. External validation is therefore needed before broader implementation.

### 4.7. Implications and Future Work

Before clinical implementation, the model should undergo independent prospective external validation in larger, contemporary multicenter cohorts representing different clinical settings and time periods. If calibration differs, recalibration of the intercept and slope may be necessary to reflect local prevalence and practice patterns. Future model updating in larger datasets should also reassess coefficient stability and may incorporate penalized estimation or additional shrinkage where appropriate. Although ridge-penalized regression in the present sensitivity analysis produced predictive performance similar to that of the standard logistic model, penalization may remain useful when the model is updated using broader datasets with greater numbers of outcome events. Future work should also evaluate implementation pathways, including integration into clinical risk calculators and the impact of risk-based prioritization on screening workload and timeliness of ROP detection [29,30,31].

## 5. Conclusions

A pragmatic prediction model for any ROP detected at the first planned ophthalmologic screening examination demonstrated good discrimination and generally satisfactory calibration after internal validation, although some overfitting remained. The model may support risk stratification among infants undergoing their first planned ophthalmologic screening examination; however, independent external validation, preferably in prospective contemporary cohorts, is required before clinical implementation.

## Figures and Tables

**Table 1:** Clinical characteristics.

Clinical Characteristics	Any ROP *n* = 50, *n* (%)	No ROP *n* = 631, *n* (%)	*p*-Value	AuROC
Male	23 (46.0)	325 (51.5)	0.467	0.528
Birth weight (grams), mean ± SD	1024.7 (±269.2)	1516.9 (±368.1)	<0.001	0.866
Gestational age (days), mean ± SD	192.3 (±16.9)	221.7 (±17.7)	<0.001	0.885
Multiple pregnancy	7 (14.0)	130 (20.6)	0.359	0.533
Respiratory distress syndrome (RDS)	39 (78.0)	358 (56.7)	0.004	0.606
Bronchopulmonary dysplasia (BPD)	28 (56.0)	147 (23.3)	<0.001	0.664
Neonatal sepsis	38 (76.0)	339 (53.7)	0.003	0.611
Neonatal anemia	29 (58.0)	196 (31.1)	<0.001	0.635
Necrotizing enterocolitis (NEC)	5 (10.0)	52 (8.2)	0.598	0.509
Oxygen exposure (days), mean ± SD	47.9 (±43.2)	14.1 (±19.4)	<0.001	0.849
Ventilator duration (days), mean ± SD	36.8 (±43.2)	8.4 (±15.4)	<0.001	0.776

ROP; retinopathy of prematurity.

**Table 2:** Multivariable logistic regression.

Predictor	Linear Model			Predictor	Quadratic Model		
	Coefficient	95% CI	*p*-Value		Coefficient	95% CI	*p*-Value
Birth weight (grams)	−0.003	−0.004, −0.001	0.004	Birth weight (grams)	−0.005454	−0.014323, 0.003414	0.228
				Birth weight squared (grams)	0.000001	−0.000002, 0.000005	0.451
Gestational age (days)	−0.060	−0.090, −0.029	<0.001	Gestational age (days)	0.024462	−0.395475, 0.444399	0.909
				Gestational age squared (days)	−0.000201	−0.001257, 0.000855	0.709
Oxygen exposure (days)	0.027	−0.009, 0.063	0.142	Oxygen exposure (days)	0.070569	0.015183, 0.125955	0.013
				Oxygen exposure squared (days)	−0.000397	−0.000800, 0.000007	0.054
Mechanical ventilation (days)	−0.019	−0.058, 0.019	0.328	Mechanical ventilation (days)	−0.032984	−0.086723, 0.020754	0.229
				Mechanical ventilation squared (days)	0.000304	−0.000128, 0.000735	0.167
Multiple pregnancy	−0.077	−1.061, 0.906	0.877	Multiple pregnancy	0.092713	−0.879607, 1.065032	0.852
RDS	−0.066	−0.887, 0.756	0.875	RDS	−0.117791	−0.962770, 0.727188	0.785
Sepsis	0.348	−0.439, 1.134	0.386	Sepsis	0.283650	−0.535419, 1.102720	0.497
NEC	−0.276	−1.402, 0.849	0.630	NEC	−0.434297	−1.639101, 0.770506	0.480
Anemia	−0.029	−0.785, 0.727	0.940	Anemia	−0.069319	−0.843739, 0.705100	0.861
BPD	−0.272	−1.072, 0.529	0.506	BPD	−0.540082	−1.384497, 0.304333	0.210
Constant	12.593	6.977, 18.209	<0.001	Constant	4.837114	−35.721470, 45.395700	0.815
Fitting parameters							*p*-value
AuROC	0.901	0.856, 0.946		AuROC	0.907	0.866, 0.948	0.200

CI, confidence interval; RDS, respiratory distress syndrome; NEC, necrotizing enterocolitis; BPD, bronchopulmonary dysplasia; AuROC, Area under the receiver operating characteristic curve.

**Table 3:** Model performance after internal validation using 1000 bootstrap resamples.

Parameters	Apparent Performance (95% CI)	Optimism-Corrected Performance (Bootstrap 95% CI)
Scaled Brier score (%)	32.4	26.1
AuROC	0.901 (0.856, 0.946)	0.883 (0.841, 0.928)
E:O ratio	1.000	1.005 (0.795, 1.247)
CITL	0.000 (−0.345, 0.345)	0.008 (−0.374, 0.385)
Calibration slope	1.000 (0.766, 1.234)	0.866 (0.606, 1.129)
Bootstrap shrinkage factor		0.866
Heuristic shrinkage		0.923

AuROC, area under receiver operating characteristic curve; E:O, expected: observed; CITL, calibration-in-the-large; C-slope, calibration-slope.

**Table 4:** Risk stratification at the sensitivity-constrained exploratory cut-off of 1.5%.

Risk Categories	Actual Any ROP		Total	Observed ROP Risk, % (95% CI)	Model-Guided Strategy
	Yes	No			
<1.50%	2 (0.3)	337 (49.5)	339 (49.8)	0.6 (0.1, 2.1)	Continue standard ROP screening schedule
≥1.50%	48 (7.0)	294 (43.2)	342 (50.2)	14.0 (10.5, 18.2)	Consider prioritized ophthalmologic assessment; proposed use requires external validation
Total	50 (7.3)	631 (92.7)	681 (100.0)		

ROP, retinopathy of prematurity; CI, confidence interval.

## Data Availability

The data underlying this article are not publicly available because they contain sensitive patient information and are subject to institutional and ethical restrictions. Requests for access to de-identified data may be directed to the corresponding author and will be considered subject to approval by the participating hospitals and the relevant ethics committees.

## Supplementary Materials

The following supporting information can be downloaded at: https://www.mdpi.com/article/10.3390/diagnostics16193207/s1, Figure S1: Comparison of receiver operating characteristic curves between the linear and quadratic model specifications; Figure S2: Center-stratified apparent receiver operating characteristic curves. Table S1; Apparent predictive performance of standard and ridge-penalized logistic regression models.

## Abbreviations

The following abbreviations are used in this manuscript:

Abbreviation	Definition
AuROC	Area under the receiver operating characteristic curve
BPD	Bronchopulmonary dysplasia
CI	Confidence interval
CITL	Calibration-in-the-large
E:O	Expected-to-observed ratio
LR+	Positive likelihood ratio
LR−	Negative likelihood ratio
NEC	Necrotizing enterocolitis
NICU	Neonatal intensive care unit
NPV	Negative predictive value
PPV	Positive predictive value
RDS	Respiratory distress syndrome
RMSE	Root mean squared error
ROC	Receiver operating characteristic
ROP	Retinopathy of prematurity
SD	Standard deviation
