# Supplementary material for: Predicting Retinopathy of Prematurity in Thailand: A Practical Model for Risk-Based Screening
Source: Diagnostics (Basel). 2026 Oct 2;16(19):3207. doi: 10.3390/diagnostics16193207 (PMC13664180; doi:10.3390/diagnostics16193207)
Supplement: Supplementary file 1 [file diagnostics-16-03207-s001.zip › diagnostics-4500505-Supplementary Table S1.pdf]

Supplementary Table S1. Apparent predictive performance of standard and ridge-penalized logistic regression models

Performance measure	Standard logistic	Ridge logistic	DeLong p-value
AuROC	0.901 (0.856–0.946)	0.900 (0.857–0.944)	0.819
CITL	0.000	0.000	
Calibration slope	1.000	1.169	
Brier score	0.046	0.047	
Scaled Brier score (%)	32.4	30.7	

DeLong p-value refers to the comparison of AuROCs.
